# Colonic Angiodysplasia with a Huge Submucosal Hematoma in the Sigmoid Colon

**DOI:** 10.1155/2016/3457367

**Published:** 2016-05-31

**Authors:** Takayuki Shimizu, Daisuke Koike, Yukihiro Nomura, Kenji Ooe

**Affiliations:** ^1^Department of Surgery, Asahi General Hospital, I-1326, Chiba 289-2515, Japan; ^2^Department of Clinical Pathology, Asahi General Hospital, I-1326, Chiba 289-2515, Japan

## Abstract

Colonic angiodysplasia (AD) with bleeding as a comorbidity in the aging population is being increasingly reported. However, to our knowledge, there is no report on colonic AD accompanied by a huge hematoma. Herein, we report a case of colonic AD with a huge submucosal hematoma. A 75-year-old man with sudden melena was referred to our hospital. Helical computed tomographic angiography (CTA) revealed bleeding from the sigmoid colon. Additionally, colonoscopy showed a huge submucosal hematoma with bleeding in the sigmoid colon. As endoscopic hemostasis was difficult, sigmoidectomy was performed. The pathological diagnosis was colonic AD. The present case indicates that colonic AD should be considered in the differential diagnosis for melena. In addition, the case shows that helical CTA, which is a noninvasive imaging modality, is useful for the diagnosis of colonic AD and is as effective as colonoscopy and angiography for diagnosis.

## 1. Introduction

The prevalence of colonic angiodysplasia (AD) has been reported to be approximately 1% [1]. However, colonic AD has been shown to be responsible for 3–40% of the cases of lower gastrointestinal tract (GIT) bleeding [2, 3]. Colonic AD is important in the differential diagnosis of melena. Although the average size of colonic AD is usually small (below 10 mm) [1], we encountered colonic AD with a huge submucosal hematoma measuring 180 × 70 mm. Herein, we report this case of colonic AD with a huge submucosal hematoma. To our knowledge, no similar case was reported previously.

## 2. Case Presentation

**Figure 1:**
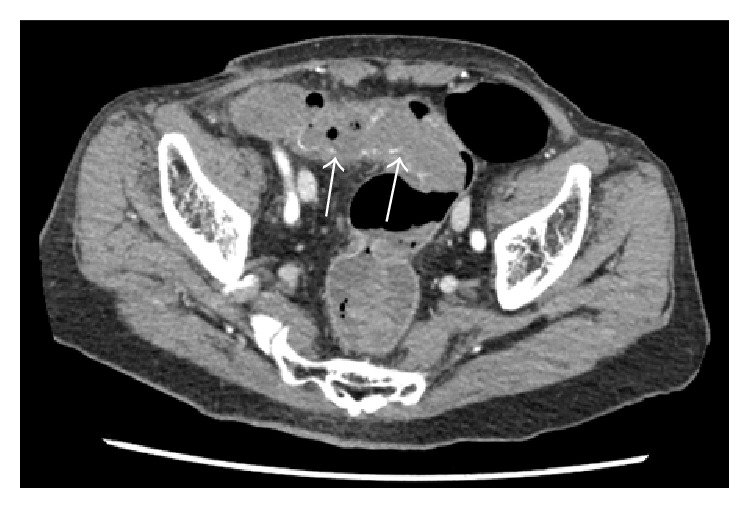
Helical computer tomographic angiography revealing the bleeding from the sigmoid colon (arrows).

**Figure 2:**
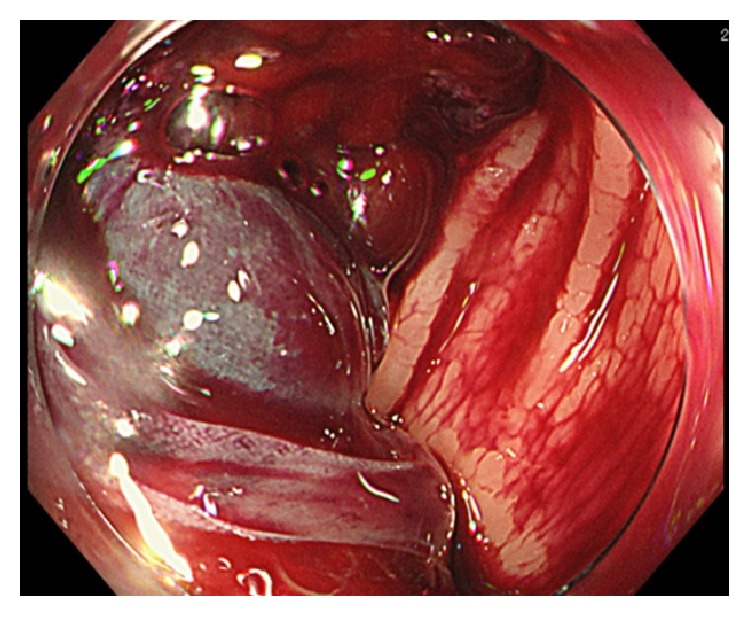
Colonoscopy showing huge submucosal hematoma in the sigmoid colon.

**Figure 3:**
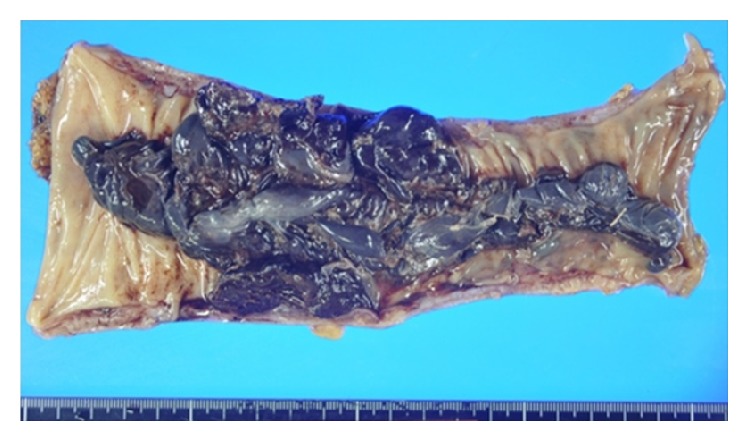
The huge submucosal hematoma in the sigmoid colon.

**Figure 4:**
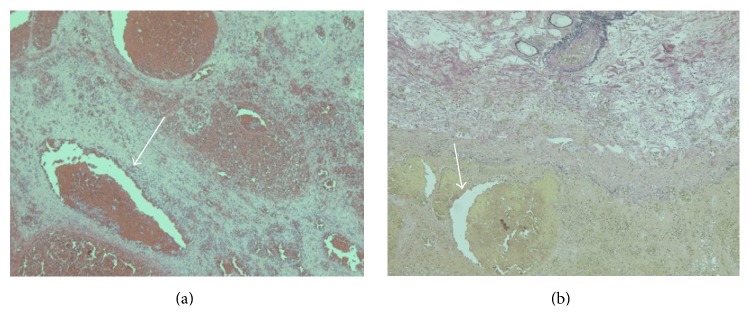
(a) Pathological finding showing an extended vessel in the submucosa of the sigmoid colon (arrow). (b) There was no elastic fiber in an extended vessel (arrow).

A 75-year-old man with sudden melena was referred to our hospital. He had a history of cardiac infarction, atrial fibrillation, high blood pressure, diabetes mellitus, and Parkinson's disease. The patient always took Bayaspirin (100 mg/day) and Xarelto (15 mg/day). The patient's systolic blood pressure was 84 mmHg, and heart rate was elevated at 105 beats/min. Physical examination showed eyelid anemia, and digital rectal examination revealed attachment of bloody stool. A blood test revealed anemia with a hemoglobin level of 10.7 g/dL. In coagulation tests, prolonged coagulation time was noted, with a prothrombin time percentage (PT%) of 68%, activated partial thromboplastin time of 42.4 s, fibrin degradation product level of 6.7 *μ*g/mL, and D-dimer level of 4.7 *μ*g/mL. Helical computed tomographic angiography (CTA) revealed an abnormal vessel in the wall of the sigmoid colon, which was visualized as a high-density area, and a hematoma in the sigmoid colon, which was visualized as a low-density area (Figure 1). Colonoscopy revealed a huge hematoma with active bleeding in the submucosal area of the sigmoid colon (Figure 2). Endoscopic hemostasis procedures, such as clipping and coagulation, could not be performed, because a huge hematoma obscured the bleed point. As blood pressure did not respond to fluid administration, an emergency sigmoidectomy was performed. During the procedure, a huge submucosal hematoma was seen through the wall of the sigmoid colon. The resected specimen showed a huge submucosal hematoma, which was 180 × 70 mm in size (Figure 3). Histopathological examination showed a submucosal extended vessel without elastic fibers in the hematoma (Figures 4(a) and 4(b)). Therefore, he was diagnosed with colonic AD. On postoperative day 7, Hartmann's operation was performed owing to an anastomotic leak. He was discharged on postoperative day 70.

## 3. Discussion

AD lesions have been defined as dilated communications between veins and capillaries [4]. Histological findings of AD include accumulation of ectatic or thin-walled veins and capillaries lined by only endothelium in the mucosa and submucosa [5], and AD can be a result in gastrointestinal bleeding owing to rupture of ectatic vessels. AD has been reported to occur in the upper and lower GIT [6]. Advanced age and comorbidities such as cardiovascular disease and chronic renal failure have been reported to be associated with colonic AD [1, 7]. In addition, advanced age and the American Society of Anesthesiologists score (≥3) were previously found to be significant risk factors for blood loss in patients with colonic AD [8]. Our patient was also elderly and had cardiovascular disease, similar to the findings in a previous report [1]. Overanticoagulation (PT < 30%) has been reported to predict the recurrence of bleeding from AD lesions [9]. Although our case was not overanticoagulation (PT = 68%), the combination of weak abnormal vessels without elastic fibers, potential weakness of tissue, and anticoagulants might induce the development of a hematoma and bleeding from the AD lesion, because he had several comorbidities such as cardiac infarction, atrial fibrillation, high blood pressure, diabetes mellitus, and Parkinson's disease, and these drugs have been shown to induce gastrointestinal bleeding [10]. It is unclear how antiplatelet and anticoagulation drugs contribute to the development of hematoma and bleeding from AD lesions. Therefore, further studies are required to clarify the relationship and mechanism.

Colonoscopy and angiography are useful for diagnosing AD and identifying the bleeding site [6]. In our case, helical CTA was useful for identifying the bleeding site. A previous study reported that helical CTA was not inferior to colonoscopy and angiography in the diagnosis of AD [11]. In our case, CTA revealed an abnormal vessel in the wall of the sigmoid colon, which was visualized as a high-density area. The high performance of CT enabled the visualization of the abnormal vessel and bleeding site in detail. It has been reported that approximately 40–60% of patients have more than one lesion of AD [12, 13]; thereby, identification of the bleeding sites is important in surgical treatment. Moreover, helical CTA can avoid local angiographic complications such as femoral hematoma and arterial dissection [11]. Thus, helical CTA is useful as a noninvasive imaging modality for the diagnosis of AD.

Therapy for AD includes endoscopic hemostasis, arterial embolization, and surgical treatment [6]. Advances in endoscopic hemostasis and arterial embolization have resulted in a marked reduction in the need for operative intervention in patients with bleeding from AD lesions [14]. However, surgical treatment should be considered for AD cases in whom fatal bleeding can occur if endoscopic hemostasis and arterial embolization are unable to control bleeding. In our case, endoscopic hemostasis was difficult, because the bleeding point was unclear. Extended arterial embolization could have caused colonic necrosis. Therefore, surgery was the most appropriate and safest therapy for our case of colonic AD with a huge submucosal hematoma, and it was successfully performed.

The present case indicates that colonic AD should be considered in the differential diagnosis of melena and that helical CTA is useful for the diagnosis of colonic AD. In conclusion, we reported a rare case of colonic AD with a huge submucosal hematoma that was successfully treated with sigmoidectomy.

## Abbreviations

AD: Angiodysplasia
ASA: American Society of Anesthesiologists
CT: Computed tomography
CTA: Computed tomographic angiography
GIT: Gastrointestinal tract.
